# The Treatment and Filling of Root Canals*Note.—This paper in full is printed in the March, 1917, *Dental Review*. It presents one side of the controversy on this subject, and we are printing an abstract of a paper by Dr. E. C. Kells, from the February *Items of Interest*, which gives a view of the other side. Both are well worth pondering.

**Published:** 1917-03

**Authors:** Edgar D. Coolidge

**Affiliations:** Chicago


### THE TREATMENT AND FILLING OF ROOT CANALS.*

BY EDGAR D. COOLIDGE, D.D.S., CHICAGO.

Dental diseases as a possible cause of systemic infection are now interesting the thought of both the medical and dental professions. The dental profession has assumed a more thoughtful attitude toward the treatment of pulpless teeth.

Now that we realize more fully the importance of properly filled root canals and the difficulty encountered in filling them, we appreciate the value of a vital pulp more than ever before. Every effort to preserve the vitality of the pulp should be made, and more frequent examinations of the teeth for incipient caries should be made.

Where bridgework is to be constructed, radiographs should be made of the teeth to be used as abutments and where there are crooked roots and other conditions present which hinder or prevent perfect root canal filling, a method of attaching should be used which will not demand removing the pulp of the tooth. We must use careful and deliberate judgment in every case, to prevent going to an extreme in our attempt to keep live pulps and having them die under fillings and crowns, or advocating crowns upon teeth which can not be properly prepared to receive a crown without endangering the life of the pulp, or attempting to place crowns on such teeth and omitting some of the necessary preparation, which later will cause an equally "unfortunate condition," chronic gingivitis.

---

NOTE.—This paper in full is printed in the March, 1917, *Dental Review*. It presents one side of the controversy on this subject, and we are printing an abstract of a paper by Dr. E. C. Kells, from the February *Items of Interest*, which gives a view of the other side. Both are well worth pondering.

Four conditions present for our consideration.

First, the vital pulp, which must be removed for some good reason. The reputation of the present methods of root canal treatment and filling will depend upon the success of our efforts with these teeth more than any others. We have the entire responsibility in these cases placed upon ourselves. We have a clear field without obstacles common to root canals having had previous operations upon them. Asepsis and thoroughness should be our motto, and nothing short of the most perfect fillings possible to make should be allowed to stand.

Second, radiographs show many canals partly filled with no evidence of infection or tissue destruction around the apex. It is a problem in many of the cases to decide whether they should be disturbed or allowed to remain. The history of the patient in regard to other teeth previously treated, and as to the general physical condition, may be helpful in determining the procedure. Where fillings or crowns are required, it is a safer method to refill the roots also as there will be less danger of future trouble. Radiographs of some of these canals show open space beyond the fillings, which is always a positive factor in determining that retreating and refilling are necessary.

Third, there are many roots of teeth showing definite rarefied areas around their apices. We have been told by those who have followed the bacteriology in these cases that it is not so much the quantity of infection present that is so dangerous, as it is the type and virulence of the bacteria. Therefore, these teeth may be as injurious to the health of the patient as those showing larger areas of absorption. Dr. Weston Price has stated that by no present method of treating can all bacteria of the rarefied area about a root end be destroyed. Should we then conclude that all teeth showing these rarefactions should be extracted? I am sure this would be too radical to recommend and would prove disastrous if carried to an extreme.

In a hospital or a bed-ridden patient case where treatment is impractical or impossible, such methods must of necessity be resorted to, but for those patients physically able to be present for treatment most of these cases can be cleared up by removing the old filling, treating and refilling. Some of these cases when followed by radiographic record, after completion of the treatment, may still show a rarefied area, and resection of the apex followed by thorough curettement should be performed.

Fourth, there are many cases showing definite areas of absorbed bone, which of course include a loss of many, if not all of the apical fibres of the peridental membrane. Many of these should be extracted for there is considerable doubt as to the cure of such large bone lesions where a considerable amount of the cementum is denuded. Regeneration of bone may take place, but a denuded and pus-soaked cementum does not afford a suitable surface for attachment or permanent apposition of new tissue. Where it is practical, the root may be retreated and refilled, followed by resection of the apex and denuded portion and the cavity curetted thoroughly.

There is no place in the field of dentistry where more careful diagnosis and keener judgment is required than in determining the condition present and prescribing the course of treatment. Many difficulties enter to confuse one in making a diagnosis from radiographs, and more than one radiograph should be taken of any doubtful area or condition. It is difficult to get a single picture to show all the roots of an upper molar to their apices, and many times the natural bone cavities, canals and foramina are confused with areas of absorption. It is hard to see the outline of some of these bone lesions where the outer plates of bone are dense and the absorption area is entirely between the two plates since the shadow of the outer plates alone will be nearly as dense as where the inner portion of bone is healthy.

*Asepsis in Root Canal Operations:* Asepsis, not antiseptics, has become the byword of modern surgery. As we look back in dentistry a few years, we recall many things of common practice which would seem ridiculous today. Who would want to have root canals treated today without the rubber dam, or have cotton wrapped upon a broach with unsterile fingers and then put into the wounded tissue in one's tooth, or to have any instrument or material used which would not be safe if used in the treatment of any wound of the body? I am sure I would not. Only a few years ago, one of the members of this society, Dr. J. P. Buckley, advocated more sanitary methods of treating root canals before this society and many arguments were made in the discussion that followed to show how impractical it all was and how unnecessary, but his good work has gone on, and we have adopted those ideas and many others. Everything should be done for asepsis in root canal operations that should be done for any surgical operation. Materials and instruments should be prepared with the greatest of care and nothing used in the canal which has been touched by the fingers. There are suitable sterilizers for materials as well as instruments, and no office should be without these necessary articles. Dry heat sterilizers are best adapted for materials such as cotton, absorbent canal points, gutta-percha points, dressings, etc., and phenol or lysol are excellent for broaches. Formaldehyd cabinets are convenient for keeping the instruments and materials in when sterilized and not in use, but they are not suitable for the entire sterilization of any of them. It is convenient and sanitary to have a tray in a formaldehyd sterilizer removable, and use it to contain all the root canal materials and instruments to be used in the operation, placing it on the operating table while the work is carried on. This is a habit that commends itself to any observing patient.

The hands should be carefully cleaned just as the operation is to be begun, but nothing should be put into the canal of the tooth which has been touched by the fingers. The field of operation should be isolated, washed with alcohol and painted with iodin, to insure against carrying any foreign material into the pulp chamber and introducing infection there.

*Technic of Cleaning and Enlarging Root Canals:* The first requirement to good results in root canal filling is to obtain sufficient access to the canal openings that they can be seen by reflected light easily and that they may be entered without working around a prominence of tooth structure. The canals that usually are small and difficult to enter are also curved as well; the root does not pass in a straight line parallel to the long axis of the tooth. These canals are the buccal canals of upper molars and mesial canals of lower molars, upper first bicuspid canals and often lower central, lateral and cuspid canals. It is often possible to eliminate at least one curve by a generous cutting into the mesiobuccal cusp and mesial marginal ridge of molars, a generous cutting buccolingually in bicuspids. When the importance of this first step has been impressed upon the mind of every operator to get the desired results there will be more than one hundred per cent. improvement in the work following.

The difficulties encountered in opening canals are so great that one's patience is often exercised to full capacity, so every detail that affords any advantage should be carried out. In extending the walls of the pulp chamber it is best to preserve the natural contour of the subpulpal wall, which usually leads toward the mouth of each canal.

The operating tray before us should contain every instrument to be used and this means everything available for this work. A number of small porcelain trays can be used to contain the short broaches and the S. S. White aseptic medicine tray is especially suitable for containing phenol and alcohol, so that each time a broach is used it can be passed into phenol, then into alcohol. This detail is a very safe precaution.

Selecting the smallest (XXX fine) smooth broach, it should be carefully worked to the end of the canal. This may be difficult, but it is very important as it gives one the direction and length of the canal. If a broach is used which will not pass to the end of the root it will bind and pack the pulp and debris farther into the canal, or else it may refuse to follow the canal and make a detour into the dentin which seldom leads back into the canal again. If a broach is first selected which will pass to the foramen and the canal enlarged throughout its entire length with the aid of thirty per cent. sulphuric acid until the next size broach can be used in the same way, much difficulty will be avoided, and more perfectly filled canals will be the result. It is a progressive performance of this same technic, using always a broach which will pass to the foramen until the canal is enlarged sufficiently to fill.

I find the Kerr pulp canal file to be one of the most useful of canal instruments. I might say that for me it has come nearer solving the problem of opening canals than any other instrument. All broaches should be used without turning them around and around in a canal. They can not follow a curved canal and be turned without breaking, so should be made to cut by drawing them back and forth like a file. It requires several hours of time spent at several appointments with a patient to clean and enlarge a small canal until it is ready to fill. Ward's sodium and potassium is sometimes very helpful in dissolving out pulp stones and calcified material, but it is dangerous to use in canals which are open to the foramen. It is an exceedingly strong caustic, and should any pass through the foramen, much harm will result. Whenever sodium and potassium is used it should be carefully neutralized with sulphuric acid and then the canal washed with sodium carbonate solution.

*Radiography:* The first time the radiograph is needed is before devitalizing to determine whether it is possible to fill the root. If the privilege were always given us of choosing the teeth to be treated much difficulty would be avoided. However, we are more often called upon to remove old fillings and treat diseased pulps than we are to remove healthy ones. Frequently a radiograph at the beginning will save difficulty and embarrassment. A tooth having had previous treatment will often have a piece of broach lodged in the canal, and it is always better to locate such obstructions before beginning an operation.

The next time the radiograph is of great value is when an obstruction of any kind is met with which prevents the broach from passing beyond a point. It may be that the broach is headed in the wrong direction, that it has failed to make a turn, and often a radiograph of a tooth with a pin in the canal will aid the operator in passing around the curve and put him on the right track again. Growing impatient and using a drill or twist broach in such cases usually increases the difficulty and lessens the possibility of getting to the foramen.

When the canal has been cleaned and enlarged it is always well to radiograph it to prove up the work and to get the measure of the length of the root as an aid in filling. The measurement wires should always be cut to extend to the full length of the crown and root, so that when the radiograph is finished the wire may be placed upon it to compare the length of the shadow and the pin to show the amount of distortion of the picture. This point is of more use to the operator than can be impressed by the simple statement. The measurement wires should be placed in safety for future reference while filling the canal. It is difficult to pass a broach into a canal as far as the foramen every time and no farther, so it is well to bend a broach to the exact length of the canal to serve as a guide.

It is needless to say that the radiograph is necessary at the completion of the canal filling, for it then becomes a matter of record to be preserved. Yet every operator will find he has occasionally failed to completely fill a canal and a refilling becomes necessary.

*Filling the Canal:* Now we are put to the final test and one upon which depends to a very large extent the future health of the apical tissue. If our operation has been performed up to this point with carefulness in regard to asepsis and the health of the apical tissue is unimpaired, we can yet spoil it all unless the canal is completely and aseptically sealed. No canal can be completely sealed by placing a long cone of guttapercha into it and attempting to pack the part remaining in the pulp chamber. The technic of filling canals with guttapercha worked out by Dr. Callahan, called the "Callahan method" from coast to coast of this country, is one by which the operator can be assured of filling all the open space in a root canal no matter how curved or tortuous it may be. In canals where the apical foramen is very small or can not be penetrated by a broach, this method of dissolving cone after cone in the chloroform rosin solution by pumping the cone up and down in the canal filled with the solution until it has become filled with a dense plastic mass, and finally packed down with pluggers, is a method unexcelled. However, many canals have open foramina through which this solution of chloropercha will be forced in increasing quantity as the operation continues, so that when completed and excess is left in the apical tissue which causes some troublesome pericementitis.

A very large per cent. of canals can and should be enlarged until a number 34 canal plugger will pass within two millimeters of the foramen. Now with the exact length of the canal taken from the measurment wire and marked upon the number 34 canal plugger, preferably by making a sharp bend at that point, the plugger is tried in the canal while the process of enlargement or curettement is going on until it will pass to within two millimeters of the apex of that canal. When this has been accomplished the canal should be dehydrated with alcohol and acetone, then slightly moistened with eucalyptol. Now we have a canal thoroughly clean and open sufficiently to allow our plugger to pass within two millimeters of the foramen. All the space to be filled beyond the plugger must be estimated from our measurements and radiograph. A short piece of guttapercha cone the proper size should be selected, about three millimeters long, and attached to the end of the plugger by heating the plugger. The space to be filled might be entirely filled by the cone; however, it is advisable in most cases to carry a small drop of thin chloropercha to the apex on a smooth broach to insure filling all the open space. The plugger carrying the three millimeter cone is passed into the canal to the market place and the cone packed into the canal.

You will readily see that this reduces the uncertainty of canal filling to a minimum, since the amount of space to be filled by guess is so small. Should the amount of guttapercha carried on the plugger be sufficient to fill to the apex, the excess can only be a portion of the drop of chloropercha used which is not enough to cause severe pericementitis following the operation. The remaining part of the canal is of little consequence if the foramen has been sealed properly by the first piece of guttapercha. The operation is continued by carrying short pieces in the same way into the canal and packing them down each time. When the canal is full it should be sealed with a hot burnisher and radiographed.

*Summary:* The consideration of the subject of root canal treatment and filling is one that must always affect the great majority of our profession. All the work can never be done by specialists in this branch of dentistry although there is a splendid opportunity for many to devote their time to it exclusively. Every operator must assume the responsibility of the root canals he fills, and as he studies this situation more he will not be satisfied with underfilled or overfilled roots, nor will he be satisfied with anything less than surgical cleanliness in this work. The health of the apical tissue must be considered of the greatest importance to a healthy individual and every operation from pulp removal to the completion of the filling of the root should be carried on with this object in view of maintaining or restoring health to this tissue. In carefully considering this thought, it will be found necessary to refuse to remove pulps from some teeth when radiographs are taken previous to operating. Caustics and strong antiseptics should be used with the greatest care if used at all. It must necessarily occupy a considerable amount of time and patience to properly clean and fill molar root canals, but it is a problem that must be met by everyone who is doing this work. There are hosts of patients coming for service of this kind daily expecting immunity from future infection. Perfectly and aseptically filled root canals are the safest protection against future alveolar abscess and in a great many cases present rarefied areas will fill up completely and give a clear radiographic record after a short time following such treatment.

